# An innovative risk-scoring system of dental procedures and safety protocols in the COVID-19 era

**DOI:** 10.1186/s12903-020-01301-5

**Published:** 2020-11-04

**Authors:** M. E. Bizzoca, G. Campisi, Lorenzo Lo Muzio

**Affiliations:** 1 Department of Clinical and Experimental Medicine, University of Foggia, Via Rovelli, 50, 71122 Foggia, Italy; 2 Department of Surgical, Oncological and Oral Sciences, University of Palermo, Palermo, Italy; 3 C.I.N.B.O. (Consorzio Interuniversitario Nazionale Per La Bio-Oncologia), Chieti, Italy

**Keywords:** Covid-19, SARS-CoV-2, Risk-scoring system, personal protective equipment (PPE)

## Abstract

**Background:**

The aim of this paper is to assess an innovative risk score for common dental procedures, based on the most recent contaminant SARS-CoV-2. After scoring the level of infection risk, safety procedures, advice and personal protective equipment (PPE) are recommended for the dental team in each dental practice.

**Methods:**

The authors of this research analysed 42 common dental procedures on the basis of known transmission risks. In increasing order, many consider the parameters leading to different risk scores for the dental team and patients for each procedure to be: direct contact with saliva (score 1), direct contact with blood (score 2), production of low levels of spray/aerosol via air–water syringes (score 3), the production of high levels of spray/aerosol from rotating, ultrasound and piezoelectric tools (score 4); and the duration of the procedure, which may increase the risk of procedures producing droplets and aerosols.

**Results:**

Using this innovative risk-scoring system, the authors classified the different dental procedures according to *low, medium* or *high* risk: *low* (1–3), *medium* (4–5), *high* (≥ 6). A safety protocol for each procedure was thereafter matched with the calculated risk level.

**Conclusions:**

The innovative risk-scoring system presented in this research permits the reclassification of dental procedures according to the infection risk level. Consequently, specific procedures, previously considered as *entry level*, will now merit revision. This paper also highlighted an effective and routine clinical tool for general dentists and oral medicine practitioners.

## Background

The Covid-19 pandemic, caused by SARS-CoV-2, has led to a global health crisis with safety and socio-economic issues. In the field of dentistry, this pandemic has highlighted the risk of infectious diseases with a clear impact on dental team and patients. In the past the most frightening pathogens were those transmissible predominantly through blood. Nowadays, and due to the SARS-CoV-2 pandemic, global security is being reconceptualised by studying prevention from infective air-borne agents, which can be diffused by the diffusion of saliva and secretions in the form of droplets and aerosol. The same phenomenon is occurring in the field of dentistry: procedures which were previously considered as trivial have increased in risk (e.g. scaling and irrigating) due to the heightened risk of spreading highly contaminated microbial aerosol. Today, the majority of the population is considered to be avoiding dental treatment except that involving pain or urgent in nature. And it can be predicted in the immediate future that these populations will fear infection from visiting the dental surgery, leading to a consequent potential increase in the price of treatments in terms of an increase in serious oral and dental diseases.

One basic principle for the recovery of any dental routine activity should be the recognized patient status of SARS-CoV-2: currently (in the emergency phase), it is advisable to perform double-phase triage (via the telephone and the surgery visit) [1]. However, in the future and in the absence of a reliable and safe test for SARS-CoV-2, the operator should consider each patient as potentially infectious, adopting individual prevention and protection measures. The main risk factors in dental practice include: a close physical relationship with the patient, coming into contact with saliva and blood, and frequent aerosol production (low level by air–water syringe or high level by rotating or ultrasonic/piezo tools).

The aim of this paper is to assess the efficacy of an innovative risk score for common dental procedures, which is based on the risk of past and principally the newest contaminant, that is, SARS-CoV-2. The authors have attempted to discern the level of infection risk relating to each dental practice, and to specify those safety procedures, advice and Personal Protective Equipment (PPE) to be deployed by the dental team. As a preliminary supposition, the authors state the requisite of recently reported fundamental postulates regarding safety protocols [1–5], with near general agreement regarding their necessity.

### Biological matrices of transmission

*Saliva* present in almost all dental procedures and a common substrate for the transmission of pathogens. Whenever possible, the patient must also wear a face covering.

*Blood* its presence during dental procedures should compel all dental staff to pay more attention to the possibility of contracting an infection.

*Aerosol* this can be said to be potentially the most dangerous and most recent difference due to SARS-CoV-2. It is produced by spray- or aerosol-producing tools, including rotating/ultrasound/piezo instruments (high level) and air–water syringes (low level). On account of their diffusing action, these tools can widely spread the saliva of a potentially infected patient, thereby exponentially increasing the risk of contamination/contagion.

*The timing of dental procedures* is also a factor to be considered. Indeed, the duration of each dental activity is related to the risk of coming into contact with pathogens and their diffusion in the environment.

### Environmental management

#### Secretary

The secretary plays an important role in ensuring protection from infection via appropriate telephone triage, effective organization of appointments in order to avoid crowding in the waiting room.

#### The treatment room

The most common chemicals suitable for surface asepsis are: chlorine, phenolic compounds, water-based, alcohol-based, and an iodophor–butoxy polypropoxy polyethoxy ethanol iodine complex [2]. All surfaces and equipment used during treatment, which are not disposable or which cannot be autoclaved [3], must be cleaned and disinfected after every patient. Any item which may be more difficult to clean must be covered with cling film, and it must be changed for each patient [4].

In addition to chemical disinfection, an UV-C ultraviolet irradiation lamp can be used. It is recommended to ventilate the rooms between patients; if this is not possible (for a minimum of 20-30 min), forced ventilation systems with High Efficiency Particulate Air (HEPA) filters must be used, paying regard to the regular replacement of filters. In order to prevent the formation of pathogens biofilm, dental unit waterlines should be rinsed for 2 min at the beginning and end of each day and for 20–30 s between patients, with a specific disinfectant agent [5]. The tubing of high-volume aspirators and saliva ejectors should be flushed regularly with water and disinfectant (sodium hypochlorite, 0.1%) between patients. In order to avoid cross-infection, the adequate sterilization of instruments is obligatory. Any item which cannot be autoclaved must be disinfected, for example by immersion in a 2% solution of glutaraldehyde. Dental waste must be disposed of in accordance with Health and Safety regulations, especially biological and pointed/sharps waste [5].

#### Dental teams and PPE

The selection of effective PPE should be based on a risk assessment and the dental procedure to be performed. Effective hand hygiene with antimicrobial soaps and before and-after dental dressing procedures must be adhered to the use of liquid soaps (or an alcohol-based hand sanitizer containing at least 60% alcohol) is recommended for a duration of 60 s. The following antimicrobial agents are also suitable: alcohol-containing cleansers, triclosan-containing products, quaternary ammonium compounds, chlorhexidine and octenidine [6].

Many consider the most useful PPE in dentistry to be:

1. face coverings, which may not, however, provide adequate respiratory protection against the small particles of aerosols; they do not prevent breath from spreading and they permit the passage of copious quantities of air to pass through the mask and to the nose and mouth.
2. a respirator, which must be worn when treating patients with respiratory infections. Particulate respirators (with filtering percentage) in use in various countries include: (a) the People’s Republic of China: II (95%), I (99%); (b) the European Union: CE-certified Filtering Face-Piece class1 (FFP1) (80%), class 2 (FFP2) (95%), or class 3 (FFP3) (99.7%); (c) Japan: 2nd class (95%), 3rd class (99.9%); and (d) the United States: National Institute for Occupational Safety and Health (NIOSH) certified N95 (95%), N99 (99%), N100 (99.7%) [7]. The powered air-purifying respirator can also be considered a standard constituent of PPE in certain situations, including aerosol-generating procedures in high risk environments.
3. goggles and face shields (personal eyeglasses and contact lenses are NOT considered as suitable eye protection) [8].
4. gowns and coveralls in Textile Non-Textile (TNT): over a dental uniform (not considered as PPE), PPE clothes must be worn and certified as suitable for biological risks according to European standards (EN 14126 and ISO 16604 (DPI) and EN 24920 (DM)).
5. gloves: gloves protect the dental operator from direct contact with mucosa and saliva. The prolonged use of gloves, washing with soap, chlorhexidine or alcohol can cause the formation of micro-perforations with increased biological risk [9]. The simultaneous use of two pairs of gloves considerably reduces the passage of pathogens through these micro-perforations [10].
6. disposable headgear and.
7. the covering of shoes.

### General recommendations

- The waiting room and treatment room must be easy to disinfect
- The patient must wear shoe coverings, hang any jacket or outer garment on a special hanger and disinfect their hands with a hydroalcoholic solution. All patients in the waiting room must be separated by a distance of not less than 2 m.
- Prior to entering the dental surgical, the patient must wear a disposable gown and headgear.
- Prior to each dental session, patients should: (1) have a 1% hydrogen peroxide 15" gargle followed by 30″ rinse, (2) not rinse with water at the end of rinsing and continue with a 0.20% chlorhexidine rinse for 60″ and a final gargle of 15″ [11] or with 0.2% povidone-iodine rinse [12, 13].
- The use of air/water syringe should be minimised by rotating/ultrasound/piezo tools, air polishing.
- Autoclavable plastic suction cannulas, with a greater suction capacity than normal disposable PVC cannulas or 2 saliva ejectors, should be used.
- Resorbable sutures are preferred.
- Refrain from touching any patient documentation/digital records and pens with used gloves.

## Materials and methods

The authors of this research analysed 42 common dental procedures as based on known SARS-CoV-2 transmission risks [1, 14]. In increasing order, many consider the parameters leading to different risk scores for the dental team and patients for each procedure to be: direct contact with saliva (score 1), direct contact with blood (score 2), production of low levels of spray/aerosol via air–water syringes (score 3), the production of high levels of spray/aerosol by use of rotating, ultrasound and piezoelectric tools (score 4); and the duration of the procedure (score 0.25 if ≤ 30 min, score 0.50 if 30–60 min, score 0.75 if ≥ 60 min). A procedure can accumulate multiple scores.

After this analysis of dental procedures, the authors classified the different dental procedures as: *low* [1–3], *medium* [4, 5] or *high* (≥ 6) risk. The duration of the procedure also plays an important role in defining the severity of the risk: the authors have provided a purely indicative timing datum since it is an operator-dependent parameter. The safety protocol for each procedure has, therefore, been, matched and proposed on the basis of its final risk score.

## Results

The results showing the risk of dental procedures according the innovative risk-scoring system are shown in Table 1. The use of PPE according to the level of risk scored, as calculated by the authors, is displayed in Table 2.

**Table 1:** Innovative risk-scoring system for 42 common dental procedures: the italics column refers to the lowest risk (score 1–3), underline relates to the intermediate (score 4–5), and Italicunderline indicates the highest risk (score ≥ 6)

Procedure	Dental specialty	Contact with saliva	Contact with blood	Use of air–water syringe (spray/aerosol production)	Use of rotating tools, ultrasonic scaler, piezo tools (spray/aerosol production)	Duration of procedure (minutes)	Grade of risk	Risk-level
*Checks in Restraint or Post-Restraint*	Orthodontics	1	0	0	0	0.50 (30–60 min)	**1.5**	Low
*Dental structure tests*	Prosthodontics	1	0	0	0	0.25 (≤ 30 min)	**1.25**	Low
*Manual reduction of dislocation of the jaw*	Gnathology	1	0	0	0	0.25 (≤ 30 min)	**1.25**	Low
*Mobile/fixed orthodontic appliance positioning*	Orthodontics	1	0	0	0	0.75 (≥ 60 min)	**1.75**	Low
*Non-invasive methods (e.g. autofluorescence, blue toluidine)*	Oral medicine	1	0	0	0	0.25 (≤ 30 min)	**1.25**	Low
*Oral rinse*	Oral medicine	1	0	0	0	0.25 (≤ 30 min)	**1.25**	Low
*Oral swab*	Oral medicine	1	0	0	0	0.25 (≤ 30 min)	**1.25**	Low
*Photo-biostimulation (Laser…)*	Oral medicine	1	0	0	0	0.25 (≤ 30 min)	**1.25**	Low
*Radiographic examination*	Diagnosis	1	0	0	0	0.25 (≤ 30 min)	**1.25**	Low
*Topical periodontal therapy*	Periodontics	1	0	0	0	0.25 (≤ 30 min)	**1.25**	Low
*Topical treatment of dental hypersensitivity and caries prophylaxis*	Hygiene and prevention	1	0	0	0	0.25 (≤ 30 min)	**1.25**	Low
*Test of night guard/bite*	Gnathology	1	0	0	0	0.25 (≤ 30 min)	**1.25**	Low
*Dental impression*	Diagnosis	1	0	0	0	0.25 (≤ 30 min)	**1.25**	Low
*Prosthetic tests, positioning and adaptation (temporary/definitive, removable/fixed)*	Prosthodontics	1	0	0	0	0.50 (30–60 min)	**1.5**	Low
*Biopsy*	Surgery	1	2	0	0	0.25 (≤ 30 min)	**3.25**	Low
*Bone graft (autogenous/biocompatible material) without rotating tools*	Surgery	1	2	0	0	0.75 (≥ 60 min)	**3.75**	Low
*Cytological sampling*	Oral medicine	1	2	0	0	0.25 (≤ 30 min)	**3.25**	Low
*Mucogingival surgery (quadrant)*	Periodontics	1	2	0	0	0.50 (30–60 min)	**3.5**	Low
*Open curettage without rotating tools (quadrant)*	Periodontics	1	2	0	0	0.50 (30–60 min)	**3.5**	Low
*Removal of cysts or small benign neoplasms*	Surgery	1	2	0	0	0.50 (30–60 min)	**3.5**	Low
*Surgical medication*	Surgery	1	2	0	0	0.25 (≤ 30 min)	**3.25**	Low
*Oral minor surgery (e.g. abscess incision, frenulectomy, frenulotomy)*	Surgery	1	2	0	0	0.25 (≤ 30 min)	**3.25**	Low
*Salivary stone removal*	Surgery	1	2	0	0	0.25 (≤ 30 min)	**3.25**	Low
*Extraction without rotating tools*	Surgery	1	2	0	0	0.50 (30–60 min)	**3.5**	Low
*Gingivectomy/gingivoplasty*	Periodontics	1	2	0	0	0.25 (≤ 30 min)	**3.25**	Low
*Endodontic treatment (1 root) with rubber dum (in subsequent appointment after access cavity)*	Endodontics	1	2	0	0	0.25 (≤ 30 min)	**3.25**	Low
*Pulp hooding, pulpotomy, pulpectomy (in subsequent appointment after access caivty) with rubber dum*	Endodontics	1	2	0	0	0.50 (30–60 min)	**3.5**	Low
Bleaching	Hygiene and prevention	1	0	3	0	0.75 (≥ 60 min)	**4.75**	Medium
Splinting	Hygiene and prevention	1	0	3	0	0.25 (≤ 30 min)	**4.25**	Medium
Visit	Diagnosis	1	0	3	0	0.25 (≤ 30 min)	**4.25**	Medium
*Tartar scaling*	Hygiene and prevention	1	2	0	4	0.50 (30–60 min)	**7.5**	High
*Extraction with rtating tools*	Surgery	1	2	0	4	0.50 (30–60 min)	**7.5**	High
*Sinus lift*	Surgery	1	2	0	4	0.75 (≥ 60 min)	**7.75**	High
*Access cavity (rotating instruments)*	Endodontics	1	2	0	4	0.25 (≤ 30 min)	**7.25**	High
*Implantology*	Surgery	1	2	0	4	0.75 (≥ 60 min)	**7.75**	High
*Open curettage (quadrant) (rotating tools)*	Periodontics	1	2	0	4	0.25 (≤ 30 min)	**7.25**	High
*Bone surgery (rotating tools)*	Periodontics	1	2	0	4	0.75 (≥ 60 min)	**7.75**	High
*Rhizectomy / rhizotomy (rotating tools)*	Periodontics	1	2	0	4	0.50 (30–60 min)	**7.5**	High
*Sealing of dental grooves*	Hygiene and prevention	1	2	0	4	0.25 (≤ 30 min)	**7.25**	High
*Apicectomy with retrograde filling*	Surgery	1	0	3	4	0.75 (≥ 60 min)	**8.75**	High
*Autologous bone harvest (rotating tools)*	Surgery	1	0	3	4	0.25 (≤ 30 min)	**8.25**	High
*Abutment tooth preparation*	Prosthodontics	1	0	3	4	0.25 (≤ 30 min)	**8.25**	High
*Odontoplasty (1 tooth)*	Gnathology	1	0	3	4	0.25 (≤ 30 min)	**8.25**	High
*Simple / complex filling using rotant tools*	Conservative	1	0	3	4	0.50 (30–60 min)	**8.5**	High

**Table 2:** Proposal of changes to personal protective equipment (PPE), according to level of risk scored for typical dental procedures

Low risk	Disposable or sterilizable headgear Surgical mask Protective goggles Disposable or sterilizable gown Double disposable latex gloves
Medium risk	**Disposable headgear** **Disposable/sterilizable visor to remove immediately** **Protective respirator (FFP2)** **Disposable gown** Double disposable latex gloves
High risk	Disposable headgear Disposable/sterilizable visor to remove immediately **FFP3 / Powered air purifying respirator (PAPR)** **Disposable protective suit** Double disposable latex gloves **Cover shoes**

PPEs in bold style are those that change from a lower risk category to a higher risk category

## Discussion

Hitherto, classifying the risk of dental procedures according to the technical difficulties and the patients’ health status has been widely discussed in the literature. The emerging situation arising from the phenomenon of COVID-19 has compelled many dental practitioners to re-classify dental procedures according to the risk of infectious contagion due to the airborne pathogens, in addition to blood pathogens. This revised risk-classification is based on greater or lesser amounts of pathogen agents, which are capable of infecting those visiting the dental surgery after direct contact with saliva and/or blood and/or aerosol/spray production. However, the grade of risk is highly influenced by the duration of contact or production of infectious materials and the related exposure time. Statistical probability studies have confirmed that, after the current and serious pandemic wave can be said to have ceased, the following should be available: repeated and intermittent social distancing, an expansion of intensive care facilities and the availability a vaccination and therapeutic practice [15, 16]. It is evident that modern working practices and modes of modern life will have to be re-evaluated to avoid the onset of new epidemics of—SARS-CoV-2 or other pathogens.

Until a few months ago, scientific knowledge and attention focused on the fact that viruses, bacteria and mycetes could be frequently present in the human mouth. Their presence in saliva may cause the direct transfer of infectious agents from infected individuals to healthy people or blood-derived contamination could transmit several infectious agents to unprotected healthy subjects [17–20]. However, it has recently been confirmed that SARS-CoV-2 can be transmitted by a positive-for SARS-CoV-2 person breathing in a room [21–25].

Thus, it can be stated that the majority of dental procedures, producing droplets and potentially highly-contaminated microbial aerosol, is extremely dangerous for all those present in the treatment room [1, 14, 23, 26] as they are generated by the typical use of ultrasonic/piezoelectric devices or rotating tools [27]. This risk is related to the number of pathogens present in the aerosol/spray, and instruments, such as rotating tools, ultrasonic scaler and piezo tools, produce a greater amount of spray and aerosol than other tools like air–water syringes. Droplets and aerosols contain a large-particle spatter of water, saliva, blood and microorganisms. This spatter travels a short distance to settle quickly, landing either on the floor (near to sanitized surfaces), the dental health care team or the patient. Spray may also contain a quantity of aerosol, which can remain airborne for extended periods of time and it may be inhaled. It has long been recognized that saliva can contain potential pathogens in quantities sufficient to infect other individuals [17, 18]. It is also known that blood-borne contamination can transmit several infectious agents to unprotected healthy subjects [19, 20]. This is, therefore, the rationale for the authors to propose the innovative COVID-19 risk-classification of dental practices, as outlined in this paper. For example, the use of rotating tools in the execution of abutment tooth preparation or the deployment of an ultrasonic scaler have become extremely dangerous since they are capable of spreading pathogens in the room through spray/aerosol. On the other hand, the manual reduction of a dislocation of the jaw or performing a dental impression are now considered by many to be less dangerous than was the case previously. With the aim of protecting all health workers, the authors suggest adhering to the security protocols (Table 2), as emanating from the recommendations of the Italian Health Ministry [28].

## Conclusion

It is the authors’ considered opinion that this innovative risk-classification can assist dental operators adhering to safety procedures and PPE provisions in order to managing dental practices in the light of the new contagious landscape. The authors are also aware that this pandemic is imposing huge changes in: the performing of telephone-triage, planning patient protocols, the number of patients/day, the timing of procedures, sanitizing the treatment room, calculating revised costs and revenues, and in the fundamental relationship of trust with their community of patients.

## Data Availability

Data sharing is not applicable to this article as no datasets were generated or analysed during the current study.

## Abbreviations

PPE: Personal protective equipment
HEPA: High efficiency particulate air
FFP: Filtering face-piece
NIOSH: National Institute for Occupational Safety and Health
TNT: Textile non-textile
